# Assessing Racial and Ethnic Discrimination in Children: A Scoping Review of Available Measures for Child Health Disparities Research

**DOI:** 10.1089/heq.2021.0008

**Published:** 2021-10-06

**Authors:** Amy S. Braddock, Allison Phad, Rachel Tabak, Shiriki Kumanyika, Shelly Johnston, Richelle Koopman, Elizabeth Prout, Amy McQueen

**Affiliations:** ^1^ Family and Community Medicine, University of Missouri School of Medicine, Columbia, Missouri, USA.; ^2^ Center for Diabetes Translation Research, Washington University in St. Louis, St. Louis, Missouri, USA.; ^3^ Brown School, Washington University in St. Louis, St. Louis, Missouri, USA.; ^4^ Community Health and Prevention, Dornsife School of Public Health, Drexel University, Philadelphia, Pennsylvania, USA.; ^5^ Pediatrics, Division of Gastroenterology, Hepatology, and Nutrition, Perelman School of Medicine, The University of Pennsylvania, Philadelphia, Pennsylvania, USA.; ^6^ Department of Medicine, School of Medicine, Washington University, St. Louis, Missouri, USA.

**Keywords:** racial discrimination, ethnic discrimination, health disparities, children and adolescents, measures, scoping review

## Abstract

**Objectives:** To characterize the availability, content, and psychometric properties of self-reported measures that assess race/ethnicity-related discrimination or psychosocial stress and have potential relevance to studies of health disparities in children and adolescents.

**Design:** Using PRISMA extension guidelines for scoping reviews, we searched Ovid Medline, CINAHL, PsychInfo, and Scopus databases from 1946 to April 20, 2020, using the search terms “stress,” “child,” “adolescents,” “discrimination,” and “psychometrics.” We limited the search to articles in English, with children and adolescents, in the United States. For each measure, we extracted information about the content, reliability, and construct validity.

**Results:** The 12 measures that met inclusion criteria assessed discrimination or stress from racial discrimination in African American children and adolescents (*n*=8), acculturative stress in Hispanic/Latino children (*n*=1), or bicultural stress in Mexican American adolescents (*n*=2), and one measure assessed both discrimination-related and acculturative stress in Hispanic/Latino children. The majority (*n*=7) articles were published between 2001 and 2010. All discrimination measures evaluated individual experiences of discrimination and one also evaluated stressfulness of discrimination and coping. The acculturative stress measures assessed general stress and immigration-related discrimination, and the bicultural stress measures evaluated many different aspects of biculturalism.

**Conclusions:** Despite the recent increased interest in the racial discrimination and stress as a contributor to racial or ethnic health disparities affecting U.S. children and adolescents, the small number of eligible measures identified and incomplete coverage of various types of racial and ethnic discrimination within and across population groups indicates a currently inadequate capacity to conduct child health disparity studies on this issue.

## Introduction

Although there are a number of reasons that racial and ethnic minority children experience health disparities, a developing body of research is exploring the direct health effects of psychosocial stress resulting from racial or ethnic discrimination. Recent events, including the deaths of George Floyd, Breonna Taylor, and others, have heightened awareness of and research interest in racial discrimination in the United States and globally.

The prevalence of African American adolescents in the United States reporting personal experiences of racial discrimination varies from 46% to 90%.^1^ Perceived racial discrimination results in a heightened stress response, suggesting a stress pathway between racial and ethnic discrimination and poor health outcomes.^2^ Studies with adults have found associations between perceived racial and ethnic discrimination and higher rates of depression and other mental health conditions, hypertension, and cardiovascular disease.^3^ A meta-analysis of 293 studies in adults found that experienced racism is associated with poorer mental, general, and physical health and that these effects are not modified by age, gender, birthplace, or education level.^4^ A systematic review of 121 studies found an association between racial discrimination and negative health outcomes, most commonly mental health outcomes, including anxiety and depression, in children and adolescents.^5^

In their 2019 policy statement, the American Academy of Pediatrics (AAP) identify experienced racism as a core determinant of child and adolescent health that pediatricians should address with their clinic patients,^6^ suggesting that measuring race/ethnic-related discrimination and the stress associated with such discrimination among children and adolescents is important for research and practice. The increased frequency of studies published in the past 15 years on the direct health impacts of stress secondary to racial and ethnic discrimination in children indicates that this is an area of increasing interest to researchers.^2–5,7–10^

Stress was defined by Lazarus and Folkman in 1984 as a situation or environment in which a person perceives that his or her resources are exceeded, resulting in psychological or psychosomatic symptoms.^11^ Most studies that try to quantify stress associated with racial discrimination use measures that evaluate personal experiences of perceived racial discrimination rather than directly measuring the experience of stress from such experiences, resulting in significant conflation of racial discrimination and stress.^12^ To effectively study the impact of stress due to experiencing racial and ethnic discrimination in children, researchers need instruments that assess concepts appropriately and have been validated for children, and the ability to evaluate the psychometric evaluation of these instruments.^3,12–14^ Moreover, standardizing methods to assess types of discrimination and stress in racial and ethnic minority children would help to strengthen research in this area by facilitating evidence synthesis.^15–17^

For this scoping review, we chose to define stress broadly to capture a diverse range of psychosocial outcomes resulting from discrimination or other experiences that are unique to racial or ethnic minority children. We included measures of racial or ethnic discrimination when discrimination was treated as a stressor. We included measures of acculturative and bicultural stress because they relate to the child's racial or ethnic identity. Acculturative stress is defined as the tension exerted on an individual by a dominant culture, as he or she engages in the presumed process of adoption of the majority group culture.^18^ In addition to the acculturative pressure to adopt the majority culture, racial and ethnic minorities can experience additional pressure to adopt or maintain their primary culture, a concept known as bicultural stress.^19^

In this scoping review, we survey the availability of self-report measures with evidence of psychometric evaluations that assess psychosocial stress perceived as resulting from a child's racial or ethnic identity, including racial or ethnic discrimination, acculturation, and bicultural stress. It is our aim to characterize what exists and identify areas for additional measure development and evaluation in this research area.

## Methods

### Search strategy and study eligibility

**FIG. 1.**
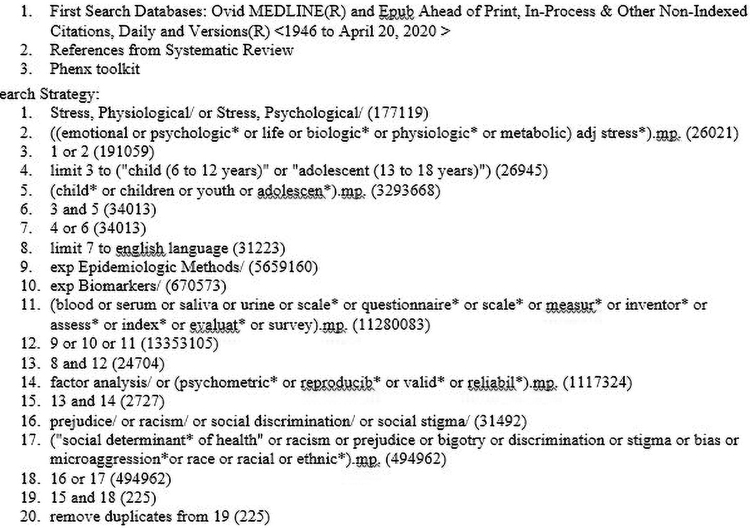
Scoping review search strategy.

Using the PRISMA extension for scoping reviews,^20^ we searched Ovid Medline, CINAHL, PsycINFO, and Scopus databases from 1946 to April 20, 2020. Search terms included synonyms for “stress,” “child,” “adolescents,” “discrimination,” and “psychometrics” (See Fig. 1 for the full search strategy). Search results were limited to articles published in English. In addition, reference lists of relevant systematic reviews were also reviewed for eligible studies.^12,21–23^ We supplemented the literature search by reviewing the PhenX toolkit,^24^ an online catalog of recommended measurement protocols, for relevant measures. Reviewed articles were not limited by study design. Eligible articles included those that focused on children or adolescents (≤18 years of age); focused on racial or ethnic minority populations as defined by the U.S. Census Bureau^25^ (for race: Black or African American, Asian, American Indian or Alaska Native, Native Hawaiian or Other Pacific Islander, or other race; for ethnicity: Hispanic or Latino); evaluated psychosocial stress that results from the participants' racial or ethnic identity including but not limited to racial discrimination/racism, acculturative stress, and bicultural stress; and reported psychometric properties for the measure being evaluated.

Studies were excluded if they only included biometric stress scales or were conducted outside the United States. Although racial and ethnic discrimination is not unique to the United States, we chose to limit our scoping review to studies conducted in the United States due to unique cultural experiences and policy-based classification systems^25^ used in the United States. In addition, the majority of racial discrimination studies to date are based in the United States.^4^

### Study selection

Two authors (A.S.B. and A.P.) reviewed titles, abstracts, and full text. Disagreements during the selection process were settled through discussion until consensus could be reached, facilitated by a third reviewer (E.P.).

### Data collection

One reviewer (A.S.B.) extracted data from the eligible articles, with accuracy of data extraction and coding of psychometric data confirmed by other authors (S.J. and A.M., respectively). Extracted data included the name of the measure, content, length, race/ethnicity and age of the study sample, and psychometric details including reliability, content, and construct validity. This study did not require institutional review board approval.

## Results

**FIG. 2.**
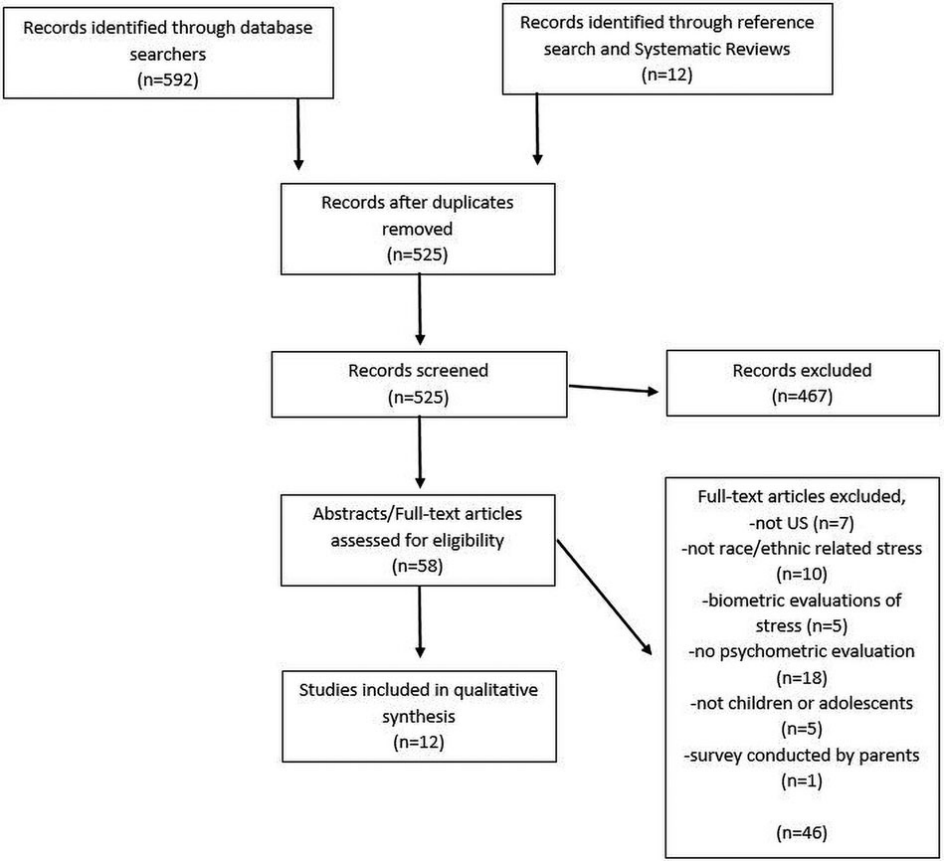
Scoping review article selection flowchart.

We identified 12 unique measures in our scoping review from the 12 articles that met the inclusion criteria. No additional articles were found using the PhenX toolkit; therefore, details are not included in the PRISMA diagram (Fig. 2). The Index of Race-Related Stress (IRRS) was evaluated in two articles,^26,27^ whereas another article^28^ evaluated two scales, the Daily Life Experiences Scale (DLE-F) and the Racism Experiences Stress Scale (EXP-STR). Only two studies were published before or during 2000,^29,30^ seven studies were published between 2001 and 2010,^26–28,31–34^ and three studies were published since 2010 (Table 1).^15,35,36^ All of the measures are available in English and three are also available in Spanish: the Hispanic Stress Inventory-Adolescent Version (HSI-A),^35^ the Mexican American Biculturalism Scale (MABS),^36^ and the Romero and Roberts scale.^33^ The age range of children participating in these studies was 8–18 years old. One scale^33^ was administered by trained teachers who read the questions out loud during regular classroom hours, and the remaining scales were self-administered. Only two articles reported the time required to complete the self-administered measure (30 min to 1 h for the IRRS^27^ and ∼40 min to complete the Adolescent Discrimination Distress Index [ADDI]).^30^ Seven measures included multiple subscales.^15,26–30,34,36^ We did not report Cronbach's α for measures with >15 items in Table 1 due to concern about interpretations of these values.^37^ Of the included α’s, five measures have Cronbach's α 0.80 to 0.89,^26–29,31,36^ four have Cronbach's α 0.70 to 0.79,^28,32,34,35^ and one has Cronbach's α 0.60 to 0.72.^30^ Most (8 studies) reported results from factor analysis^15,26,27,30–32,34–36^ and 10 studies included a description of how the scale was developed to support content validity.^15,26,27,29–33,35,36^

**Table 1.** Summary of Measures

		Sample characteristics						Construct validity		
Category, First Author (Pub year)	Instrument name	n	Race/ethnicity	Ages (years)	Content Development	Structure	Scales (# items)	Alpha	Related measures	Known groups test
Racial/ethnic discrimination										
Clark (2004)	Everyday Discrimination Scale (EDS)	120	African American	14–18	M	1	EDS (9)	0.87	Internalizing, externalizing symptoms	Ever > never experienced racism
LaFont (2018)	Child Perceived Discrimination Questionnaire (CPDQ)	163	African American	9–11	Y	4	Exclusion by children (3) Threats/harassment by children (5) Exclusion by adults (3) Threats/harassment by adults (5)		Depression, emotion regulation, externalizing symptoms, risky behaviors	Black > White (discrimination by adults subscale only)
Pachter (2010)	Perceptions of Racism in Children and Youth (PRaCY)	227	Latino and African American	8–13 14–18	Y	1	Ten final items for each age group (not all 10 were the same)	0.78 for each age group	Depressive symptoms, anxiety (for age 8–13)	Scores differed by race/ethnic group
Scott (2005)	Daily Life Experiences Scale (DLE-F), Racism Experiences Stress Scale (EXP-STR)	71	African American	14–18	M		DLE-F (10) EXP-STR (10)	0.77 0.88	Racial centrality & externalizing (both), internalizing (EXP-STR only)	
Seaton (2003)	Index of Race-Related Stress (IRRS)	324	African American	13–18	M	3	Individual racism (10) Collective\institutional racism (13) Cultural racism (9)	0.87 0.84 0.82		
Seaton (2006)	Index of Race-Related Stress (IRRS)	258	African American	13–19	M	3	Individual racism (10) Collective\institutional racism (13) Cultural racism (9)	0.89 0.94 0.89	Adapted Perceived Racism Scale (PRS) subscales	
Fisher (2000)	Adolescent Discrimination Distress Index (ADDI)	177	African American, Hispanic, East or South Asian, non-Hispanic White	13–19	Y	3	Institutional discrimination (6) Educations Discrimination (4) Peer discriminations (5)	0.72 0.60 0.60	Racial Bias Preparation Scale (RBPS), Rosenberg Self-Esteem Inventory	Non-Hispanic White < all other groups; 12th graders >9–11th graders for institutional discrimination
Cervantes (2012)	Hispanic Stress Inventory-Adolescent Version (HSI-A)	1651	Hispanic	10–20	Y	8	One hundred sixty items reduced to 72; 8 factors (not detailed here)	0.64–0.85	Children's Depression Inventory (CDI), Youth Self Report (YSR) psychopathologies	
Acculturative stress										
Chavez (1997)	Societal, Attitudinal, Familial, and Environmental Acculturative Stress Scale for Children (SAFE-C)	1651	Latino & White	8–10	Y		General social stressors (16) Acculturation stressors (20)			Higher scores in Latino versus White
Racial/ethnic discrimination and acculturative stress										
Suarez-Morales (2007)	Acculturative Stress Inventory for Children (ASIC)	139	Hispanic	M=10.47 SD=0.56	N	2	Perceived Discrimination (8) Immigration-related Stress (4)	0.79 0.72	Daily Hassles Questionnaire, Revised Children's Manifest Anxiety Scale	Scores differed by race/ethnic group
Bicultural stress										
Basilio (2014)	Mexican American Biculturalism Scale (MABS)	316	Mexican American	M=15.87 SD=0.43	Y	3	Bicultural comfort (9) Bicultural facility (9) Bicultural advantages (9)	0.85 0.81 0.86	Language use and pressures, perceived discrimination, ethnic identity	
Romero (2003)		881	Mexican	11–15	Y		Twenty items		Language use, perceived SES, depressive symptoms, self-esteem	Immigrant versus U.S.-born

Details of content development provided: N=No, Y=Yes, M=modified existing measure; structure: empirical support for number of factors/subscales; construct validity: significant correlations with other related measures noted (not included are intercorrelations between subscales).

M, mean; SD, standard deviation.

### Measures of experiences of racial or ethnic discrimination

We identified eight scales that evaluate racial or ethnic discrimination: Everyday Discrimination Scale (EDS),^31^ Child Perceived Discrimination Questionnaire (CPDQ),^15^ Perceptions of Racism in Children and Youth (PRaCY),^32^ combined DLE-F and the EXP-STR,^28^ IRRS,^26,27^ ADDI,^30^ and the HSI-A.^35^

A modified version of the 9-item EDS developed by Forman et al.^38^ asked respondents to indicate how often they experienced specific events in their day-to-day life because of their race.^31^ Sample items are “treated with less courtesy” and “called names.” Response options range from 1 “almost every day” to 6 “never.” This measure was evaluated with African American children of ages 14–18 years. The Cronbach's α was 0.87, with a split-half reliability of 0.83. The authors reported a one component structure based on principal components analysis with varimax rotation, and reported positive associations with internalizing and externalizing symptoms supporting construct validity (Table 1).

The 16-item CPDQ was modified from existing items for use with children.^15^ The authors describe their qualitative research to support the content validity of the new measure that evaluates everyday experiences of discrimination and includes four subscales—exclusion by children, threat/harassment by children, exclusion by adults, and threat/harassment by adults. Sample items include “other children called you names” and “a teacher or other adult treated you unfairly.” Responses range from 1 “never” to 5 “very often.” It was evaluated in 9–11-year-old African Americans. In their study, the authors reported support for the four hypothesized subscales using confirmatory factor analysis and support construct validity by reporting positive associations with several measures of emotion and risky behavior (Table 1).

To develop the 10-item PRaCY, respondents evaluated the prevalence, attribution, and emotional and coping responses to 23 different discriminatory situations including “someone was rude to you” and “people assume you're not smart or intelligent.” The measure was initially developed using qualitative research methods and a final instrument was evaluated with Latino and African American children of ages 8–18 years. Results of confirmatory factor analysis and differential item functioning found support for a single factor with items that were not biased by age, gender, or ethnicity. The measure's Cronbach's α was 0.78. Some support for construct validity was reported as positive correlations with depressive and anxiety symptoms for the 8–13 years age group.^32^

The DLE-F and the EXP-STR measures were developed from items in a unpublished Racism and Life Experiences Scale.^39^ These measures assess perceived discrimination (including the experience of macroaggressions in everyday life attributable to race or racism) and discrimination distress or the stressfulness of such experiences. Each measure includes 10 items. Respondents rate the frequency of experiencing each event from 0 “never” to 4 “all the time,” and for each experience, rate the degree of perceived stress evoked from 0 “no stress” to 4 “extremely stressful.” The measures were evaluated with 14–18-year-old African Americans. Cronbach's α was 0.77 for the DLE-F and 0.88 for the EXP-STR (Table 1). The authors also reported support for construct validity for both measures with positive correlations with racial centrality and externalizing symptoms.^28^

The IRRS was adapted from a 46-item measure developed to evaluate the stress of daily experiences of racial discrimination experienced by African American adults.^40^ In two studies, Seaton evaluated 32 items of the IRRS in a sample of 13–19-year-old African Americans and found support for three subscales that evaluate individual, institutional/collective, and cultural racism, with Cronbach's α of 0.89, 0.94, and 0.89, respectively (Table 1). Sample items include “security people have followed you while shopping in some stores” and “you have observed the police treat Whites/non-Blacks with more respect than they do Blacks.” Response options for the IRRS range from 0 “this has never happened to me” to 4 “event happened and I was extremely upset.” Expected associations were found between factors and with subscales in an adapted Perceived Racism Scale supporting construct validity.^27^

The ADDI is a 15-item measure designed to evaluate discrimination in a variety of races and ethnicities (African American, Hispanic, Asian, non-Hispanic White). Students 13–19 years were asked whether they had experienced different types of discrimination based on racial/ethnic groups and the degree to which (on a 5-point scale) it upset them. Examples include “you were hassled by police” or “you were called racially insulting names.” The authors found support for three subscales determined using principal components analysis. The subscales evaluate institutional, educational, and peer discriminations with Cronbach's α of 0.72, 0.60, and 0.60, respectively (Table 1). The test–retest reliability for the three subscales were 0.76, 0.53, and 0.75, respectively (Table 1).^30^

The HSI-A evaluates life events stress exposure and its appraisal. Content validity is supported by the qualitative research methods used to develop the original 160 life events evaluated in a study with Hispanic children and young adults of ages 10–20 years.^35^ After deleting poor performing items, results of exploratory factor analysis supported the interpretation of eight subscales reflecting family economic stress, acculturation gaps stress, culture and education stress, immigration-related stress, discrimination stress, family immigration stress, community and gang violence stress, and family drug-related stress. The Cronbach's α for the eight subscales ranged from 0.64 to 0.85 and support for construct validity was found with positive associations with measures of depression and psychopathology (Table 1).

### Measures of acculturative stress

The Societal, Attitudinal, Familial, and Environmental Acculturative Stress (SAFE) scale was modified for children (SAFE-C): it has 36 items related to general and acculturative stress. It was evaluated with Latino 8–10-year-old children. Sample stress items include “I worry that other kids won't like me” (general) and “people think I am shy, when I really just have trouble speaking English” (acculturative). Response options range from 0 “does not apply to me,” 1 “doesn't bother me” to 5 “bothers me a lot.” No evidence of factorial or construct validity was reported.^29^

### Measures of racial or ethnic discrimination and acculturative stress

The Acculturative Stress Inventory for Children (ASIC) included 12 items from the 36-item SAFE-C and evaluates discrimination specific to acculturative stress in two subscales supported by exploratory factor analysis reflecting perceived discrimination and immigration-related stress.^34^ In a sample of 5th grade (mean age 10.47 years) Hispanic students, support for internal consistency reliability of each factor and for construct validity was reported (Table 1).

### Measures of bicultural stress

The MABS includes 27 items with 3 subscales: bicultural comfort, bicultural facility, and bicultural advantages (9 items each); 5-point response scales are used but labels vary by subscale.^36^ Qualitative methods were used to develop the items and quantitative analysis supports the internal consistency reliability, factorial validity, and construct validity for a sample of Mexican American adolescents (mean age of 16 years) and their mothers and fathers.^36^ Tests of measurement invariance suggest that the MABS can be used to compare scale scores across age groups, gender, and languages. This was the only study we reviewed that reported factorial invariance.^36^

The Romero and Roberts^33^ measure is a 20-item scale adapted from previous bicultural and adult stress scales, literature reviews, qualitative data, and pilot study results. Although no evidence of factorial validity was reported, an aggregate score was found to be associated with measures of self-esteem, depression symptoms, language use, and perceived socioeconomic status (Table 1).

## Discussion

Our goal with this scoping review was to review the status of published measures with evidence of psychometric evaluation that assess psychosocial stress resulting from a child's racial or ethnic identity. Such measures are essential for the ability to understand the role of this type of stress as contributors to racial/ethnic health disparities.

We identified 12 relevant measures designed to assess discrimination or stress in children or adolescents; the focus of the measures differed according to the population group: racial discrimination in African American children and adolescents, acculturative stress in Hispanic/Latino children, and bicultural stress in Mexican American adolescents. Only one instrument allowed for overlap in the experiences of discrimination and acculturative stress within the same group. The HSI-A evaluates both ethnic discrimination and acculturative stress in Hispanic adolescents.

We found no measures that evaluate acculturative or bicultural stress in African American children or adolescents, despite the well-documented presence of both types of stress in African American adults.^41–43^ The pressure to conform to a majority or dominant White culture may be similar or perhaps even greater during the formative adolescent years. African American adolescents, especially male adolescents, often experience strong acculturative pressure to conform to majority society but are often unable to, due to assumptions of irreconcilable differences. At the same time, they may feel pressure to maintain African American linguistic and cultural expectations,^44^ compounding the bicultural stress and the mental health distress that result.^45^

Despite the presence of 12 measures with at least some evidence of psychometric evaluation, we found significant deficiencies in the literature. There is a need for more studies that evaluate measures of different types of racial and ethnic discrimination, including institutional and cultural racism, as well as for other racial minorities including Asian American and Native Americans. We found only one measure (ADDI) designed to be used in Asian American populations and none for Native Americans. Furthermore, we could not find evidence of psychometric evaluation for commonly used adult stress scales, such as the perceived stress scale, in racial or ethnic minority children.^46^

Although most of the measures evaluate general stress and/or experiences of racial/ethnic discrimination or mistreatment, only three measures (PRaCY, DLE-F/EXP-STR, and ADDI) sufficiently link the two concepts and evaluate the child's appraisal of their experience. Testing hypothesized models of stress as a mediator of perceived discrimination on health outcomes will require more measures of the perceived stressfulness of events, not simply the frequency such events are experienced. Validating children's ability to self-report the stressfulness of specific events in addition to reliably reporting their exposure to specific events is critical for describing the prevalence across samples and assessing change over time.

Although some measures have more thorough psychometric evaluations than others, more studies of metric properties are needed in different populations on currently available instruments. In addition, new instrument development is needed to begin to fill these identified gaps. The quality of the psychometric evaluation is also important and should be considered when making direct comparison of metric properties across studies. For example, some measures were developed from a single study using exploratory factor analysis, which requires replication in future studies using confirmatory methods. While many studies reported mean differences by race/ethnic group or nativity, only one study tested measurement invariance that is required for such comparisons lest we infer meaningful differences where measurement differences may be evident. This is especially critical for studies investigating the prevalence and impact of stress due to racial/ethnic identity and the variability across racial/ethnic groups. Finally, we chose to only report Cronbach's α when support for the unidimensionality of the scale was also reported.^37,47^

There is some debate about the most meaningful way to evaluate racial and ethnic discrimination, including objective versus subjective assessments.^48^ We included only self-reported subjective measures of perceived racial/ethnic discrimination and intentionally excluded studies that included biomarkers of stress such as cortisol. One could argue that biomarkers could offer a more objective measurement of stress, as compared with self-reported scales, but these tests require more expense and technical expertise to use and may not be practical for some studies.

In addition, self-reported measures may provide more insight into a child's response to perceived discrimination or racism, acculturation, or biculturalism, because this experience is inherently subjective and depends on a multitude of factors including coping, motivation to ignore prejudice-related events, and inclination to report prejudice events, all of which might mediate a physiological stress response.^48^

A 2010 systematic review identified 24 measures of perceived racial discrimination in adults.^21^ In comparison, our scoping review yielded only eight measures for racial discrimination in African American, Hispanic, and Asian children only. Unfortunately, many of the studies that evaluated the impact of racial discrimination in children were not conducted with measures with any psychometric evaluation.^5^ In their adult review, Bastos et al.^21^ also found multiple measures that assess not just experiences of discrimination, but also related constructs including institutional racism, and emotional and behavioral coping responses, which were present in only three measures (PRaCY, DLE-F/EXP-STR, and ADDI) in our child scoping review. This suggests that the field of stress related to ethnicity and race in children is still developing. The use of qualitative and mixed methods research may help advance the field and develop additional measures.

## Conclusion

In conclusion, despite the presence of 12 measures with evidence of psychometric evaluation that evaluate stress from racial discrimination in African American, Hispanic, and Asian children and adolescents, acculturative stress in Hispanic/Latino children, and bicultural stress in Mexican American adolescents, additional measures are needed to evaluate different types of racial discrimination including institutional and cultural racism. We also found a paucity of measures appropriate for use with other racial minorities including Asian Americans and Native Americans. In addition, more testing of the psychometric properties of the available measures in additional samples, and with more extensive psychometric evaluations, is also needed.

There were several limitations to our scoping review. We did not include conference abstracts or unpublished measures in our review, increasing the risk of publication bias. However, our aim was to provide a review of measures with psychometric evaluation in the published/public domain that can be used by researchers, and not provide an exhaustive review of all related measures being used in empirical research.

We also only included publications that were available in English (although three measures were also available in Spanish) and conducted only in the United States. This provides for a relatively clear interpretation within the U.S. context but may limit application to other settings, particularly settings in which explicit use of race-specific language and acknowledgment of racism (e.g., as opposed to “ethnicity” or ethnic biases) is less common or consistent than in the United States.

The breadth of operational definitions of stress that were evaluated with these measures provide insight into the complexity of defining and measuring stress secondary to racial and ethnic discrimination. The use of these measures with demonstrated reliability and validity for children and adolescent racial/ethnic minority populations can help to bring standardization and rigor to this field, which is necessary to accurately quantify stress secondary to racial and ethnic discrimination and its impact on health in children and adolescents.

Different conceptual frameworks for racial discrimination, acculture, and bicultural stress will continue to require different measures to evaluate them. Only by measuring and quantifying these factors can we understand the scope of the problem and begin to design and evaluate interventions that address it. Until we are able to better understand and quantify this often-overlooked stressor, our understanding of health disparities in children will be incomplete. Although more research is needed, this review provides a basis for health researchers to begin measuring and mitigating the health effects of racism and discrimination.

## Abbreviations Used

AAP: American Academy of Pediatrics
ADDI: Adolescent Discrimination Distress Index
ASIC: Acculturative Stress Inventory for Children
CPDQ: Child Perceived Discrimination Questionnaire
DLE-F: Daily Life Experiences Scale
EDS: Everyday Discrimination Scale
EXP-STR: Racism Experiences Stress Scale
HSI-A: Hispanic Stress Inventory-Adolescent Version
IRRS: Index of Race-Related Stress
MABS: Mexican American Biculturalism Scale
PRaCY: Perceptions of Racism in Children and Youth
SAFE: Societal, Attitudinal, Familial, and Environmental Acculturative Stress
SD: standard deviation

## References

[1] Cooper SM, McLoyd VC, Wood D, et al. Racial discrimination and the mental health of African American adolescents. In: Handbook of Race, Racism, and the Developing Child. Hoboken, NJ: John Wiley & Sons, Inc., 2008, pp. 278–312.

[2] Pascoe EA, Smart Richman L. Perceived discrimination and health: a meta-analytic review. Psychol Bull. 2009;135:531–554. 19586161 10.1037/a0016059 PMC2747726

[3] Williams DR, Lawrence JA, Davis BA. Racism and health: evidence and needed research. Annu Rev Public Health. 2019;40:105–125. 30601726 10.1146/annurev-publhealth-040218-043750 PMC6532402

[4] Paradies Y, Ben J, Denson N, et al. Racism as a determinant of health: a systematic review and meta-analysis. PLoS One. 2015;10:e0138511. 26398658 10.1371/journal.pone.0138511 PMC4580597

[5] Priest N, Paradies Y, Trenerry B, et al. A systematic review of studies examining the relationship between reported racism and health and wellbeing for children and young people. Soc Sci Med. 2013;95:115–127. 23312306 10.1016/j.socscimed.2012.11.031

[6] Trent M, Dooley DG, Douge J, et al. The impact of racism on child and adolescent health. Pediatrics. 2019;144:e20191765. 31358665 10.1542/peds.2019-1765

[7] Gee GC, Walsemann KM, Brondolo E. A life course perspective on how racism may be related to health inequities. Am J Public Health. 2012;102:967–974. 22420802 10.2105/AJPH.2012.300666 PMC3483932

[8] Dominguez TP, Dunkel-Schetter C, Glynn LM, et al. Racial differences in birth outcomes: the role of general, pregnancy, and racism stress. Health Psychol. 2008;27:194–203. 18377138 10.1037/0278-6133.27.2.194 PMC2868586

[9] Pachter LM, Coll CG. Racism and child health: a review of the literature and future directions. J Dev Behav Pediatr. 2009;30:255–263. 19525720 10.1097/DBP.0b013e3181a7ed5a PMC2794434

[10] Slopen N, Williams DR. Discrimination, other psychosocial stressors, and self-reported sleep duration and difficulties. Sleep. 2014;37:147–156. 24381373 10.5665/sleep.3326 PMC3865350

[11] Lazarus RS, Folkman S. Stress, Appraisal, and Coping. New York: Springer, 1984.

[12] Paradies Y. A systematic review of empirical research on self-reported racism and health. Int J Epidemiol. 2006;35:888–901. 16585055 10.1093/ije/dyl056

[13] Paradies Y. A systematic review of empirical research on self-reported racism and health. In: Race, Ethnicity, and Health: A Public Health Reader, 2 ed. Edited by LaVeist TA, Isaac LA. San Francisco, CA: Jossey-Bass, 2013, pp. 105–138.

[14] Salihu HM, Salinas-Miranda AA, King LM, et al. Racism, psycho-social stress, and health-related quality of life. Int J MCH AIDS. 2020;9:73–76. 32123630 10.21106/ijma.339 PMC7031886

[15] LaFont SR, Brondolo E, Dumas AK, et al. The development and initial validation of the Child Perceived Discrimination Questionnaire. Int J Cult Ment Health. 2018;11:208–219. 31768191 10.1080/17542863.2017.1356337 PMC6876697

[16] Jones SCT, Anderson RE, Gaskin-Wasson AL, et al. From “crib to coffin”: navigating coping from racism-related stress throughout the lifespan of Black Americans. Am J Orthopsychiatry. 2020;90:267–282. 32105125 10.1037/ort0000430 PMC8807348

[17] Varcoe C, Browne A, Blanchet Garneau A. Beyond stress and coping: the relevance of critical theoretical perspectives to conceptualising racial discrimination in health research. Health Sociol Rev. 2019;28:245–260.

[18] Berry JW, Kim U. Acculturation and mental health. In: Health and Cross-Cultural Psychology: Toward Applications. Thousand Oaks, CA: Sage Publications, Inc., 1988, pp. 207–236.

[19] Romero AJ, Martinez D, Carvajal SC. Bicultural stress and adolescent risk behaviors in a community sample of Latinos and non-Latino European Americans. Ethn Health. 2007;12:443–463. 17978943 10.1080/13557850701616854

[20] Tricco AC, Lillie E, Zarin W, et al. PRISMA extension for scoping reviews (PRISMA-ScR): checklist and explanation. Ann Intern Med. 2018;169:467–473. 30178033 10.7326/M18-0850

[21] Bastos JL, Celeste RK, Faerstein E, et al. Racial discrimination and health: a systematic review of scales with a focus on their psychometric properties. Soc Sci Med. 2010;70:1091–1099. 20122772 10.1016/j.socscimed.2009.12.020

[22] Gagnon AJ, Tuck J, Barkun L. A systematic review of questionnaires measuring the health of resettling refuge women. Health Care Women Int. 2004;25:111–149. 14766429 10.1080/07399330490267503

[23] Yoo HC, Pituc ST. Assessments of perceived racial stereotypes, discrimination, and racism. In: APA Handbook of Testing and Assessment in Psychology: Vol. 2 Testing and Assessment in Clinical and Counseling Psychology. Edited by Geisinger KF, Bracken BA, Carlson JF, et al. Washington, DC: American Psychological Association, 2013, pp. 427–451.

[24] Hamilton CM, Strader L, Pratt J, et al. The PhenX Toolkit: get the most from your measures. Am J Epidemiol. 2011;174:253–260. 21749974 10.1093/aje/kwr193 PMC3141081

[25] U.S. Census Bureau. Race & Ethnicity. 2017. Available at https://www.census.gov/mso/www/training/pdf/race-ethnicity-onepager.pdf Accessed June 1, 2019.

[26] Seaton EK. An examination of the factor structure of the Index of Race-Related Stress among a sample of African American adolescents. J Black Psychol. 2003;29:292–307.

[27] Seaton EK. Examination of a measure of racial discrimination among African American adolescents. J Appl Soc Psychol. 2006;36:1414–1429.

[28] Scott LD Jr., House LE. Relationship of distress and perceived control to coping with perceived racial discrimination among black youth. J Black Psychol. 2005;31:254–272.

[29] Chavez DV, Moran VR, Reid SL, et al. Acculturative stress in children: a modification of the SAFE scale. Hisp J Behav Sci. 1997;19:34–44.

[30] Fisher CB, Wallace SA, Fenton RE. Discrimination distress during adolescence. J Youth Adolesc. 2000;29:679–695.

[31] Clark R, Coleman AP, Novak JD. Brief report: initial psychometric properties of the Everyday Discrimination Scale in black adolescents. J Adolesc. 2004;27:363–368. 15159094 10.1016/j.adolescence.2003.09.004

[32] Pachter LM, Szalacha LA, Bernstein BA, et al. Perceptions of Racism in Children and Youth (PRaCY): properties of a self-report instrument for research on children's health and development. Ethn Health. 2010;15:33–46. 20013438 10.1080/13557850903383196 PMC2891186

[33] Romero AJ, Roberts RE. Stress within a bicultural context for adolescents of Mexican descent. Cultur Divers Ethnic Minor Psychol. 2003;9:171–184. 12760328 10.1037/1099-9809.9.2.171

[34] Suarez-Morales L, Dillon FR, Szapocznik J. Validation of the Acculturative Stress Inventory For Children. Cultur Divers Ethnic Minor Psychol. 2007;13:216–224. 17638478 10.1037/1099-9809.13.3.216

[35] Cervantes RC, Fisher DG, Cordova D, et al. The Hispanic Stress Inventory—Adolescent Version: a culturally informed psychosocial assessment. Psychol Assess. 2012;24:187–196. 21942232 10.1037/a0025280 PMC3391962

[36] Basilio CD, Knight GP, O'Donnell M, et al. The Mexican American Biculturalism Scale: bicultural comfort, facility, and advantages for adolescents and adults. Psychol Assess. 2014;26:539–554. 24548151 10.1037/a0035951 PMC4041836

[37] Cortina JM. What is coefficient alpha? An examination of theory and applications. J Appl Psychol. 1993;78:98–104.

[38] Forman TA, Williams DR, Jackson JS. Race, place, and discrimination. In: Perspectives on Social Problems. Edited by Gardner C. Bingley, NY: Emerald Publishing Limited, 1997, pp. 231–261.

[39] Harrell SP, Merchant MA, Young SA. In: *Psychometric Properties of the Racism and Life Experiences Scale (RaLES)*. Chicago, IL: Annual Convention of the American Psychological Association, 1997

[40] Utsey SO, Ponterotto JG. Development and validation of the Index of Race-Related Stress (IRRS). J Couns Psychol. 1996;43:490–501.

[41] Anderson LP. Acculturative stress: a theory of relevance to black Americans. Clin Psychol Rev. 1991;11:685–702.

[42] Utsey SO, Walker RL, Dessources N, et al. A psychohistorical analysis of the African American bicultural experience. In: Handbook of Racial-Cultural Psychology and Counseling, Vol 2: Training and Practice. Hoboken, NJ: John Wiley & Sons, Inc., 2005, pp. 410–426.

[43] Berhe ZB. Examining the Relationship between Bicultural Stress, Mental Well-Being, Perceived Social Support, And Education Among People of African Descent [Ph.D.]. Ann Arbor, Seton Hall University, 2015.

[44] Neal-Barnett A. Being Black: a new conceptualization of acting White. In: *Forging Links: African American Children Clinical Developmental Perspectives*. Edited by Neal-Barnett A, Contreras J, Kerns K. Westport, CT: Greenwood Publishing Group, Inc., 2001

[45] Walker RL. Acculturation and acculturative stress as indicators for suicide risk among African Americans. Am J Orthopsychiatry. 2007;77:386–391. 17696667 10.1037/0002-9432.77.3.386

[46] Cohen S, Kamarck T, Mermelstein R. A global measure of perceived stress. J Health Soc Behav. 1983;24:385–396. 6668417

[47] Panayides P. Coefficient alpha: interpret with caution. Eur J Psychol. 2013;9:687–696.

[48] Meyer IH. Prejudice as stress: conceptual and measurement problems. Am J Public Health. 2003;93:262–265. 12554580 10.2105/ajph.93.2.262 PMC1447727

